# Association between smoking cessation and depressive symptoms according to cessation duration, pack-years, and tobacco product type: a nationwide cross-sectional study in Korea

**DOI:** 10.3389/fpubh.2026.1755259

**Published:** 2026-03-26

**Authors:** Jungyeon Kim, Eunsu Lee, Daehee Hwang, Ah Jung Ko, Eun-Cheol Park

**Affiliations:** 1 Premedical Courses, Yonsei University College of Medicine, Seoul, Republic of Korea; 2 Department of Public Health, Graduate School, Yonsei University, Seoul, Republic of Korea; 3 Institute of Health Services Research, Yonsei University, Seoul, Republic of Korea; 4 Department of Preventive Medicine, Yonsei University College of Medicine, Seoul, Republic of Korea

**Keywords:** cessation duration, depression, former smokers, pack-years, smoking cessation

## Abstract

**Background:**

Depression, a growing global mental health concern, has multifactorial causes and is frequently associated with cigarette smoking. There is a persistent misconception that quitting smoking worsens depressive symptoms. This study examined the association between smoking cessation and depressive symptoms and whether this relationship varies according to cessation duration, cumulative tobacco exposure, and tobacco product type.

**Methods:**

A total of 231,469 participants from the 2024 Korea Community Health Survey were included. The Patient Health Questionnaire-9 was used to evaluate depressive symptoms. Participants’ smoking status (never, current, or former smoker), cessation duration, cumulative tobacco exposure, and primary tobacco product type were determined based on the survey responses. Multiple logistic regression analyses were conducted to examine associations between smoking cessation and depressive symptoms in this cross-sectional dataset.

**Results:**

Compared with never-smokers, both former and current smokers showed elevated odds of depressive symptoms. However, former smokers showed lower odds than current smokers (men: aOR = 1.18 vs. 1.36; women: aOR = 1.64 vs. 1.96). Among former smokers, those who had quit for less than a year showed the highest odds of depressive symptoms, with a gradual decline as cessation duration increased. In men, higher pack-years corresponded to greater odds of depressive symptoms. Dual former smokers of conventional and electronic cigarettes demonstrated significantly higher odds of depressive symptoms than exclusive conventional cigarette users.

**Conclusion:**

Smoking cessation was associated with lower odds of depressive symptoms compared with current smoking, although former smokers still showed higher odds of depressive symptoms than never smokers. These findings should be interpreted cautiously given the cross-sectional design, which precludes causal inference and raises the possibility of reverse causality. Among former smokers, women, recent quitters, individuals with higher cumulative exposure, and dual users may represent subgroups vulnerable to depressive symptoms.

## 1 Introduction

Depression is a common, debilitating, and serious mental disorder that constitutes a major public health crisis worldwide. It affects approximately 5% of the adult population globally, is a leading cause of disability, and significantly diminishes quality of life (1). Within this global landscape, the Republic of Korea faces a particularly acute and escalating mental health challenge. National survey data revealed an alarming and sustained increase in the prevalence of major depressive disorder (MDD) from 1.6% in 2001 to 3.1% in 2011, while more recent studies have shown a further increase from 4.7% in 2014 to 7.0% in 2018 (2). This global and national context underscores the urgency of developing effective strategies for the prevention and management of depressive disorders.

The risk factors for depression are complex and multifactorial and include a family history of depression, early life trauma, chronic stress, and certain personality traits, such as high neuroticism. Sociodemographic variables are consistently correlated with depression, with higher rates of depression typically observed in women, unmarried individuals, and those with a lower income or educational attainment (2). While these factors provide a crucial understanding of vulnerability for depression, many are not readily modifiable (3).

Consequently, research and public health interventions increasingly focus on the pivotal role of modifiable lifestyle behaviors. Among the lifestyle factors, substance use, particularly tobacco smoking, plays a central role. Individuals with depression are significantly more likely than the general population to smoke, meet the criteria for nicotine dependence, find it more difficult to quit, and are more prone to relapse after a cessation attempt. This relationship is observed across the sexes, although some evidence suggests that depression may have a greater impact on smoking cessation outcomes in women (3).

A substantial barrier to smoking cessation, particularly among individuals with mental health conditions, is the widespread but mistaken belief that quitting exacerbates depression and anxiety. However, multiple systematic reviews and meta-analyses have found that smoking cessation is associated with significant reductions in symptoms of depression, anxiety, and stress, as well as improvements in mood and overall psychological quality of life (4). The magnitude of this improvement is not trivial; the effect size for the reduction in depressive symptoms following cessation (standardized mean difference ranging from −0.25 to −0.30) is comparable to or even larger than the effect sizes reported for antidepressant medications used to treat mood and anxiety disorders (4). This finding reframes smoking cessation from a simple lifestyle choice to a potent evidence-based mental health treatment.

The relationship between smoking and depression is increasingly conceptualized as bidirectional (5). Individuals with depressive symptoms may initiate or maintain smoking with the intent to regulate their mood through nicotine-induced dopaminergic and serotonergic effects, which is explained by the self-medication hypothesis. The self-medication hypothesis proposes that individuals use substances such as nicotine or alcohol to alleviate psychological and mental distress (6). Furthermore, according to negative reinforcement mechanisms, the repeated experience of temporary relief from negative affect through smoking may strengthen nicotine dependence, thereby making cessation attempts increasingly difficult (7). According to stress-coping theory, individuals may choose smoking as a behavioral strategy to cope with psychological stress, and such repeated use may further reinforce nicotine dependence through negative reinforcement mechanisms (8, 9).

In this study, we sought to provide a detailed and clinically relevant understanding of the relationship between smoking cessation and depressive symptoms by conducting subgroup analyses of former smokers in a large representative sample of Korean adults. We examined how this association varies according to the duration of smoking cessation, cumulative lifetime tobacco exposure (quantified in pack-years), and the primary type of tobacco product used prior to quitting.

## 2 Materials and methods

### 2.1 Study population and data

This study used data from the 2024 Korea Community Health Survey (KCHS), an annual nationwide survey conducted by the Korea Disease Control and Prevention Agency. The KCHS is an official national statistical survey in South Korea and produces regional-level health statistics to support the implementation of community health programs. The target population comprised adults aged 19 years or older residing within the jurisdictions of 255 community health centers nationwide. In this study, a multi-stage stratified probability sampling design was used to ensure regional representation.

**Figure 1:**
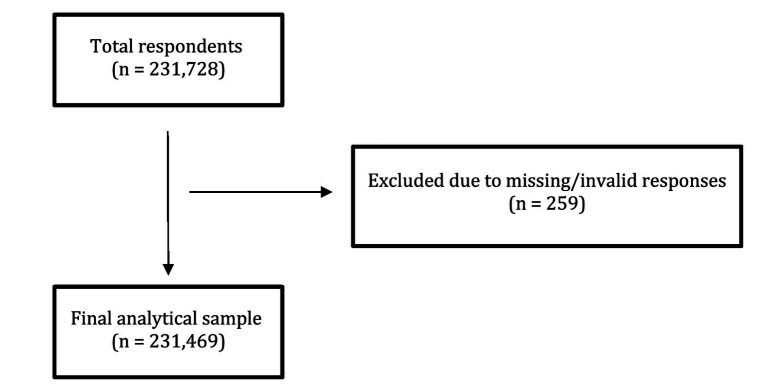
Flowchart of participant inclusion and exclusion.

Among the 231,728 respondents, those with missing or invalid responses to key variables (smoking status, depressive symptoms, and sociodemographic covariates) were excluded, yielding a final analytical sample of 231,469 participants. Because the KCHS is a cross-sectional survey, temporal ordering between smoking cessation and depressive symptoms could not be established. In addition, individuals who successfully quit smoking may differ systematically from those who continue smoking, including having fewer depressive symptoms or better overall health profiles, raising the possibility of healthy quitter bias. A flowchart summarizing participant inclusion and exclusion is presented in Figure 1. This study was conducted in accordance with the principles outlined in the Declaration of Helsinki.

### 2.2 Measures

#### 2.2.1 Patient health questionnaire-9

Depressive symptoms were assessed using the Patient Health Questionnaire-9 (PHQ-9). The PHQ-9 is a widely validated 9-item self-report instrument that corresponds to the diagnostic criteria for MDD found in the DSM-IV (10). Owing to its brevity and reliability, it is widely used in both clinical and epidemiological research. Participants rate the frequency of symptoms experienced during the previous two weeks on a 4-point scale ranging from 0 (“not at all”) to 3 (“nearly every day”), with total scores ranging from 0 to 27.

In this study, participants were classified as having depressive symptoms when the total PHQ-9 score was ≥5. Although this cutoff reflects mild depressive symptoms rather than clinically diagnosed major depression, it was selected to capture a broader spectrum of clinically relevant psychological distress in the general population. This definition was supported by a validation study conducted in the Korean population, in which the PHQ-9 demonstrated excellent discriminatory power [area under the curve (AUC) = 0.909, *p* < 0.001] and identified a score of 5 as the optimal cutoff, yielding a sensitivity of 89.9% and specificity of 84.1% (11).

To examine whether the findings were consistent across different levels of symptom severity, sensitivity analyses were conducted using established PHQ-9 severity thresholds (≥5 for mild, ≥10 for moderate, and ≥20 for severe depressive symptoms).

#### 2.2.2 Smoking and smoking cessation-related variables

**Figure 2:**
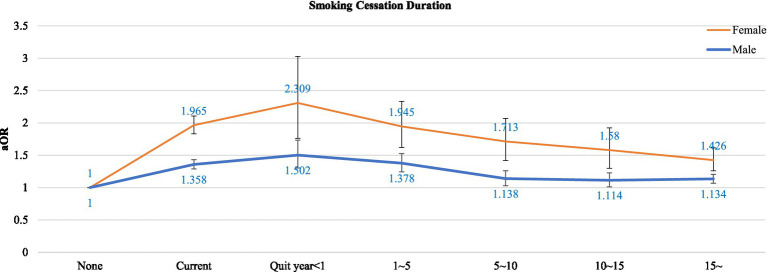
Adjusted odds ratios for depressive symptoms among former smokers by duration of smoking cessation.

Smoking status was defined based on lifetime smoking experience and current smoking behavior. Participants were first asked whether they had ever used any type of tobacco product during their lifetime, including conventional cigarettes and electronic cigarettes (e-cigarettes) Figure 2.

Those who answered “*yes*” were asked additional questions about each product type. For conventional cigarettes, participants responded to a question on their lifetime smoking amount using the following options: (1) less than five packs, (2) five packs or more, or (3) never smoked. For e-cigarettes, responses were dichotomized as “*yes*” or “*no.*”

Participants who had used any tobacco product were asked about their current smoking status, with possible responses: (1) smoke every day, (2) smoke occasionally, or (3) used to smoke but do not smoke currently.

Based on these responses, the participants were categorized into three groups: (1) never smokers, those who reported smoking fewer than five packs of conventional cigarettes in their lifetime and had never used any type of e-cigarette (HTP or liquid type); (2) current smokers, those who currently used at least one type of cigarette (conventional or e-cigarette); (3) former smokers, those who had previously used at least one type of cigarette but were not currently smoking.

Former smokers were further divided into subgroups according to smoking cessation duration, pack-years, and product type to explore differential associations within this group.

Smoking cessation duration was assessed based on the question of former conventional cigarette smokers: “*How long has it been since you quit smoking conventional cigarettes?*” Possible responses included (1) less than 1 year, (2) 1–5 years, (3) 5–10 years, (4) 10–15 years, (5) 15–20 years, and (6) 20 years or more. For the analysis, these responses were combined into five categories: <1 year, 1–5 years, 5–10 years, 10–15 years, and ≥15 years.

Pack-years for former conventional cigarette smokers were calculated as follows: (number of cigarettes smoked per day × years smoked) ÷ 20. The participants were categorized into four groups: <10, 10–19, 20–29, and ≥30 pack-years. These cut-off values were selected with reference to previous epidemiologic studies that examined dose–response patterns using similar pack-year categories, and to ensure adequate numbers in each category for stable estimates (12).

Information on pack-years and cessation duration for e-cigarette users was not available in the survey and thus excluded from the analysis.

The previously used type of tobacco products was defined according to the specific kind of tobacco products that participants had used and were classified into three categories: (1) conventional cigarette-only users, (2) e-cigarette-only users, and (3) dual users (both conventional cigarettes and e-cigarettes).

#### 2.2.3 Covariates

The analysis was adjusted for three main categories of covariates: sociodemographic characteristics, health-related behaviors, and health status factors.

The sociodemographic covariates included age, educational attainment, household income, marital status, and employment status. Age was categorized into six groups (19–29, 30–39, 40–49, 50–59, 60–69, and ≥70 years). Educational attainment was classified into two groups: “high school diploma or less” and “college graduate or higher.” Marital status and employment status were both dichotomized as “married” or “unmarried” and “employed” or “unemployed,” respectively.

Health-related behaviors included alcohol consumption, physical activity, and sleep duration. Alcohol consumption was classified into three groups: non-drinkers, low-risk drinkers, and high-risk drinkers, with high-risk drinking defined according to the KCHS criteria (≥7 drinks for men or ≥5 drinks for women per occasion, ≥2 times per week) (13). Physical activity was defined based on the KCHS definition for regular walking, with participants categorized as “active” (≥30 min/day) or “inactive” (<30 min/day). The average daily sleep duration was calculated using a weighted average: ([average weekday sleep hours] × 5 + [average weekend sleep hours) × 2]/7. Based on the weighted average, the participants were classified into three groups: “short sleepers” (<7 h), “adequate sleepers” (7–9 h), and “long sleepers” (>9 h). This categorization is based on standard recommendations for healthy adult sleep duration (14).

Finally, we adjusted for health status covariates, including obesity, presence of chronic diseases, and perceived stress level. Obesity was included as a binary variable (“obese” or “non-obese”). The presence of chronic diseases was defined as “absent” (neither hypertension nor diabetes mellitus) or “present” (having either hypertension or diabetes mellitus). Perceived stress was assessed using a single self-reported question asking participants to rate the level of stress they experienced in daily life. Responses were recorded on a four-point scale and were categorized into two groups (yes vs. no) for the analysis. Perceived stress was included as a covariate because it may act as a common correlate of both smoking behavior and depressive symptoms. However, we acknowledge that stress may also lie on the pathway linking smoking and depressive symptoms; therefore, its role should be interpreted cautiously.

### 2.3 Statistical analysis

All analyses were stratified according to sex. A formal interaction test between sex and smoking status was performed to assess potential effect modification. The interaction term was statistically significant (*F* = 55.03, *p* < 0.0001), indicating that the association between smoking status and depressive symptoms differed by sex. Therefore, all subsequent analyses were conducted separately for men and women. Chi-square tests were used to analyze and compare variables. To examine the relationship between smoking cessation and depressive symptoms, we conducted multiple logistic regression analysis after adjusting for all covariates. Subgroup analyses were performed to assess the combined associations among smoking cessation, other covariates, and depression.

To further explore the heterogeneity among former smokers, additional subgroup analyses were conducted to evaluate the associations between the type of previously used tobacco product, cumulative smoking exposure (pack-years), duration of smoking cessation, and the prevalence of depressive symptoms.

Furthermore, depressive symptoms were classified into mild, moderate, and severe categories based on the PHQ-9 scores, and the association between smoking cessation and the degree of depressive severity was examined using multinomial logistic regression analysis.

The results were presented as odds ratios (ORs) and 95% confidence intervals (CIs) to compare the prevalence of depressive symptoms. All analyses accounted for sample stratification, clustering, and post-stratification weights using a complex survey design. Analyses were conducted using SAS software (version 9.4; SAS Institute, Cary, NC, USA), and *p*-values <0.05 were considered statistically significant.

## 3 Results

A total of 231,469 participants (106,035 men and 125,434 women) were included in the analysis. General characteristics of the study population stratified by sex are presented in Table 1. Among them, 13.8% of men and 20.9% of women reported experiencing depressive symptoms, as defined by a PHQ-9 score ≥5. For both sexes, the proportion of participants with depressive symptoms increased in the following order: never smokers, former smokers, and current smokers. Among men, the prevalence increased from 11.4% among never smokers to 13.3% among former smokers and 16.1% among current smokers. Among women, the corresponding proportions were 19.8%, 32.0%, and 38.7%, respectively.

**Table 1:** Socioeconomic and health-related characteristics of study participants according to depressive symptoms.

Variables		Male (*N* = 106,035)				*p*-value	Female (*N* = 125,434)				*p*-value
		PHQ-9<5		PHQ-9≥5			PHQ-9<5		PHQ-9≥5		
		N	(%)	N	(%)		N	(%)	N	(%)	
Total (*N* = 231,469)		91,441	(86.2)	14,594	(13.8)		99,164	(79.1)	26,270	(20.9)	
Smoking status						<.0001					<.0001
	Never-smoker	25,624	(88.6)	3,284	(11.4)		93,421	(80.2)	23,071	(19.8)	
	Current smoker	30,657	(83.9)	5,895	(16.1)		3,074	(61.3)	1,942	(38.7)	
	Former smoker	35,137	(86.7)	5,412	(13.3)		2,665	(68.0)	1,256	(32.0)	
Age						<.0001					<.0001
	20-29	8,968	(87.9)	1,231	(12.1)		8,530	(80.7)	2,038	(19.3)	
	30-39	10,137	(84.9)	1,799	(15.1)		9,764	(78.5)	2,678	(21.5)	
	40-49	13,240	(86.4)	2,088	(13.6)		14,058	(82.9)	2,894	(17.1)	
	50-59	17,883	(88.3)	2,378	(11.7)		19,530	(83.5)	3,871	(16.5)	
	60-69	21,638	(88.2)	2,898	(11.8)		23,426	(82.4)	4,994	(17.6)	
	70-	19,575	(82.3)	4,200	(17.7)		23,856	(70.9)	9,795	(29.1)	
Household income						<.0001					<.0001
	Low	29,152	(80.6)	7,012	(19.4)		37,807	(73.0)	13,984	(27.0)	
	Moderate	21,783	(88.4)	2,853	(11.6)		20,448	(81.7)	4,581	(18.3)	
	High	39,053	(89.7)	4,497	(10.3)		39,198	(84.3)	7,274	(15.7)	
Education						<.0001					<.0001
	≥college	54,148	(84.6)	9,830	(15.4)		65,423	(76.9)	19,637	(23.1)	
	<college	37,264	(88.7)	4,757	(11.3)		33,703	(83.6)	6,624	(16.4)	
Marital status						<.0001					<.0001
	Married	62,016	(88.3)	8,235	(11.7)		61,257	(82.5)	12,977	(17.5)	
	Not married	29,411	(82.2)	6,358	(17.8)		37,898	(74.0)	13,291	(26.0)	
Employment status						<.0001					<.0001
	Yes	68,523	(88.5)	8,885	(11.5)		59,252	(82.5)	12,556	(17.5)	
	No	22,913	(80.1)	5,709	(19.9)		39,906	(74.4)	13,713	(25.6)	
Alcohol status						<.0001					<.0001
	Non-Drinker	33,432	(83.8)	6,465	(16.2)		63,020	(77.7)	18,115	(22.3)	
	Low-Risk Drinker	41,678	(88.4)	5,462	(11.6)		32,306	(82.6)	6,806	(17.4)	
	High-Risk Drinker	16,329	(86.0)	2,666	(14.0)		3,836	(74.0)	1,348	(26.0)	
Physical activity						<.0001					<.0001
	No	42,436	(84.2)	7,987	(15.8)		48,748	(75.8)	15,566	(24.2)	
	Yes	48,994	(88.1)	6,606	(11.9)		50,405	(82.5)	10,703	(17.5)	
Sleep duration						<.0001					<.0001
	< 7 Hours	38,880	(82.9)	8,004	(17.1)		42,846	(73.1)	15,795	(26.9)	
	7-9 Hours	50,721	(89.4)	6,004	(10.6)		54,502	(85.0)	9,651	(15.0)	
	> 9 Hours	1,781	(75.5)	577	(24.5)		1,745	(68.9)	786	(31.1)	
Obesity						0.1098					<.0001
	No	57,078	(87.5)	8,131	(12.5)		73,007	(80.0)	18,298	(20.0)	
	Yes	34,358	(84.2)	6,460	(15.8)		26,157	(76.6)	7,972	(23.4)	
Chronic disorder						<.0001					<.0001
	No	57,078	(87.5)	8,131	(12.5)		65,545	(81.8)	14,586	(18.2)	
	Yes	34,358	(84.2)	6,460	(15.8)		33,608	(74.2)	11,683	(25.8)	
City						<.0001					<.0001
	Metropolitan areas	22,239	(84.3)	4,136	(15.7)		24,217	(78.0)	6,842	(22.0)	
	Small and medium-sized cities	29,149	(86.2)	4,678	(13.8)		31,892	(79.0)	8,465	(21.0)	
	Rural areas	40,053	(87.4)	5,780	(12.6)		43,055	(79.7)	10,963	(20.3)	
Stress						<.0001					<.0001
	No	78,450	(91.4)	7,356	(8.6)		85,199	(86.3)	13,526	(13.7)	
	Yes	12,981	(64.2)	7,236	(35.8)		13,950	(52.3)	12,739	(47.7)	

Table 2 presents the results of multiple logistic regression analysis examining the association between smoking status and depressive symptoms. After adjusting for covariates, participants of both sexes who were current smokers exhibited a higher likelihood of depressive symptoms [adjusted odds ratio (aOR) = 1.36, 95% CI: 1.29–1.43 in men; aOR = 1.96, 95% CI: 1.83–2.10 in women]. In addition, former smokers (aOR = 1.18, 95% CI: 1.12–1.25 in men; aOR = 1.64, 95% CI: 1.52–1.78 in women) showed a higher likelihood of depressive symptoms than never-smokers but a lower likelihood than current-smokers.

**Table 2:** Adjusted odds ratios for depressive symptoms by smoking status and covariates.

Variables		Depressive symptoms (PHQ-9 ≥5)			
		Male		Female	
		aOR	95% CI	aOR	95% CI
Smoking status					
	Never-Smoker	1.00		1.00	
	Current Smoker	1.36	(1.29–1.43)	1.96	(1.83–2.10)
	Former Smoker	1.18	(1.12–1.25)	1.64	(1.52–1.78)
Age					
	20-29	1.00		1.00	
	30-39	1.59	(1.45–1.74)	1.36	(1.26–1.47)
	40-49	1.40	(1.28–1.54)	1.02	(0.95–1.10)
	50-59	1.10	(1.00–1.20)	0.99	(0.92–1.06)
	60-69	1.11	(1.02–1.22)	0.92	(0.85–0.99)
	70-	1.59	(1.44–1.75)	1.43	(1.33–1.55)
Household income					
	Low	1.00		1.00	
	Middle	0.66	(0.62–0.70)	0.76	(0.72–0.79)
	High	0.59	(0.56–0.62)	0.70	(0.67–0.73)
Education					
	≥college	1.00		1.00	
	<college	1.20	(1.15–1.26)	1.18	(1.13–1.23)
Marital status					
	Married	1.00		1.00	
	Not married	1.50	(1.44–1.57)	1.35	(1.30–1.39)
Employment status					
	Yes	1.00		1.00	
	No	1.63	(1.55–1.71)	1.48	(1.43–1.53)
Alcohol status					
	Non-drinker	1.00		1.00	
	Low-risk drinker	0.79	(0.75–0.82)	0.92	(0.88–0.95)
	High-risk drinker	0.87	(0.83–0.93)	1.14	(1.06–1.24)
Physical activity					
	No	1.00		1.00	
	Yes	0.77	(0.74–0.80)	0.72	(0.70–0.74)
Sleep duration					
	< 7 Hours	1.63	(1.57–1.69)	1.86	(1.80–1.91)
	7-9 Hours	1.00		1.00	
	> 9 Hours	1.95	(1.75–2.18)	1.84	(1.67–2.03)
Obesity					
	No	1.00		1.00	
	Yes	0.98	(0.94–1.02)	1.04	(1.00–1.07)
Chronic disorder					
	No	1.00		1.00	
	Yes	1.21	(1.16–1.27)	1.24	(1.19–1.28)
City					
	Metropolitan areas	1.00		1.00	
	Small and medium-sized cities	0.82	(0.78–0.87)	0.91	(0.87–0.94)
	Rural areas	0.68	(0.65–0.71)	0.78	(0.75–0.81)
Stress					
	No	1.00		1.00	
	Yes	6.37	(6.11–6.63)	6.11	(5.91–6.31)

aOR, adjusted odds ratio; CI, confidence interval.

After stratifying the participants according to their characteristics, subgroup analyses were performed using a multiple logistic regression model (Table 3). When stratified by age group, the aORs for depressive symptoms among former smokers tended to decrease with age in men, whereas in women, higher aORs were observed among younger participants. These findings should be interpreted cautiously, as the study was not designed to formally evaluate sex-by-age interactions. When stratified by alcohol consumption, former smokers who reported drinking had lower odds of depressive symptoms than nondrinkers. This pattern may reflect underlying differences in social or health characteristics between drinkers and nondrinkers rather than a protective effect of alcohol per se, and therefore should be interpreted with caution.

**Table 3:** Subgroup analyses of the association between smoking status and depressive symptoms.

Variables	Depressive symptoms (PHQ-9 ≥5)										
	Male						Female–				
	Never-smoker	Current smoker		Former smoker			Never-smoker	Current smoker		Former smoker	
		aOR	95% CI	aOR	95% CI			aOR	95% CI	aOR	95% CI
Age											
	20-29	1.00	1.35	(1.17–1.56)	0.97	(0.75–1.26)	1.00	2.69	(2.27–3.18)	1.77	(1.38–2.28)
	30-39	1.00	1.22	(1.08–1.39)	1.09	(0.92–1.28)	1.00	2.01	(1.68–2.41)	1.66	(1.37–2.00)
	40-49	1.00	1.30	(1.14–1.49)	1.08	(0.93–1.26)	1.00	2.10	(1.78–2.49)	1.66	(1.39–1.99)
	50-59	1.00	1.46	(1.26–1.68)	1.31	(1.13–1.52)	1.00	1.66	(1.40–1.95)	1.85	(1.53–2.24)
	60-69	1.00	1.34	(1.18–1.53)	1.10	(0.97–1.25)	1.00	1.97	(1.68–2.32)	1.58	(1.29–1.92)
	70-	1.00	1.18	(1.05–1.33)	1.19	(1.08–1.30)	1.00	1.22	(0.99–1.49)	1.47	(1.24–1.75)
Household income											
	Low	1.00	1.36	(1.25–1.48)	1.16	(1.07–1.26)	1.00	1.94	(1.76–2.15)	1.61	(1.43–1.80)
	Middle	1.00	1.34	(1.20–1.50)	1.21	(1.07–1.36)	1.00	1.79	(1.54–2.07)	1.50	(1.26–1.78)
	High	1.00	1.32	(1.21–1.44)	1.17	(1.07–1.29)	1.00	2.09	(1.84–2.38)	1.78	(1.55–2.04)
Education											
	≥college	1.00	1.31	(1.21–1.42)	1.11	(1.02–1.22)	1.00	2.27	(1.97–2.61)	1.73	(1.50–1.99)
	<college	1.00	1.40	(1.30–1.50)	1.22	(1.14–1.31)	1.00	1.90	(1.75–2.06)	1.60	(1.46–1.76)
Marital status											
	Married	1.00	1.27	(1.18–1.37)	1.15	(1.08–1.23)	1.00	1.91	(1.70–2.14)	1.68	(1.50–1.88)
	Not Married	1.00	1.41	(1.31–1.53)	1.19	(1.09–1.30)	1.00	1.99	(1.82–2.18)	1.60	(1.43–1.78)
Employment status											
	Yes	1.00	1.26	(1.18–1.34)	1.10	(1.03–1.18)	1.00	1.85	(1.69–2.03)	1.53	(1.37–1.71)
	No	1.00	1.46	(1.32–1.61)	1.26	(1.15–1.37)	1.00	2.06	(1.83–2.30)	1.76	(1.57–1.97)
Alcohol status											
	Non-Drinker	1.00	1.36	(1.25–1.48)	1.28	(1.19–1.38)	1.00	1.96	(1.76–2.18)	1.82	(1.63–2.03)
	Low-Risk Drinker	1.00	1.32	(1.22–1.43)	1.05	(0.97–1.15)	1.00	1.96	(1.74–2.19)	1.53	(1.35–1.75)
	High-Risk Drinker	1.00	1.30	(1.12–1.51)	1.11	(0.94–1.30)	1.00	1.98	(1.66–2.36)	1.37	(1.08–1.74)
Physical activity											
	No	1.00	1.41	(1.31–1.52)	1.20	(1.12–1.30)	1.00	1.85	(1.68–2.03)	1.67	(1.50–1.85)
	Yes	1.00	1.29	(1.20–1.39)	1.16	(1.07–1.25)	1.00	2.09	(1.89–2.32)	1.61	(1.43–1.82)
Sleep duration											
	0-6(hr)	1.00	1.38	(1.28–1.48)	1.15	(1.07–1.24)	1.00	1.93	(1.75–2.12)	1.65	(1.49–1.84)
	7-9(hr)	1.00	1.36	(1.26–1.47)	1.22	(1.12–1.32)	1.00	2.05	(1.84–2.29)	1.67	(1.48–1.88)
	9-(hr)	1.00	1.05	(0.78–1.41)	1.18	(0.88–1.57)	1.00	1.58	(1.11–2.24)	1.14	(0.76–1.73)
Obesity											
	No	1.00	1.35	(1.26–1.45)	1.22	(1.14–1.31)	1.00	2.03	(1.87–2.21)	1.67	(1.52–1.84)
	Yes	1.00	1.34	(1.23–1.45)	1.11	(1.02–1.21)	1.00	1.75	(1.54–1.98)	1.57	(1.37–1.80)
Chronic disorder											
	No	1.00	1.39	(1.30–1.48)	1.20	(1.12–1.29)	1.00	2.07	(1.91–2.25)	1.64	(1.49–1.81)
	Yes	1.00	1.25	(1.14–1.36)	1.11	(1.03–1.21)	1.00	1.70	(1.49–1.94)	1.65	(1.44–1.88)
City											
	Metropolitan areas	1.00	1.39	(1.26–1.53)	1.21	(1.09–1.34)	1.00	1.88	(1.66–2.14)	1.63	(1.41–1.87)
	Small and medium-sized cities	1.00	1.40	(1.28–1.54)	1.14	(1.04–1.26)	1.00	2.14	(1.90–2.41)	1.71	(1.50–1.96)
	Rural areas	1.00	1.29	(1.18–1.41)	1.19	(1.10–1.29)	1.00	1.81	(1.61–2.04)	1.57	(1.37–1.79)
Stress											
	No	1.00	1.41	(1.32–1.51)	1.23	(1.15–1.32)	1.00	1.98	(1.81–2.18)	1.69	(1.52–1.86)
	Yes	1.00	1.28	(1.18–1.39)	1.10	(1.00–1.20)	1.00	1.93	(1.73–2.14)	1.59	(1.40–1.80)

aOR, adjusted odds ratio; CI, confidence interval.

Figure 1 shows the results of the multiple logistic regression analysis after subgrouping former smokers according to the duration of smoking cessation. Those who had quit smoking within 1 year exhibited higher aORs for depressive symptoms than current smokers (aOR = 1.50, 95% CI: 1.30–1.73 in men; aOR = 2.31, 95% CI: 1.76–3.03 in women). Nonetheless, the aORs progressively decreased with longer cessation duration. Among men, the ORs were significantly lower than those of current smokers after 5 years of smoking cessation (aOR = 1.14, 95% CI: 1.03–1.26).

Table 4 presents the results of the subgroup analyses among former smokers stratified by pack-years and types of tobacco products. Former smokers who had used both conventional and e-cigarettes (dual users) exhibited higher aORs (aOR = 1.51, 95% CI: 1.33–1.72 in men; aOR = 1.91, 95% CI: 1.56–2.33 in women) for depressive symptoms than those who had used only conventional cigarettes (aOR = 1.18; 95% CI: 1.11–1.25 in men; aOR = 1.61; 95% CI: 1.48–1.76 in women). Individuals who used only e-cigarettes showed no statistically significant association, likely because of the small sample size of this group.

**Table 4:** Adjusted odds ratios for depressive symptoms among former smokers by pack-years, and primary tobacco product type.

Variables		Depressive symptoms (PHQ-9 ≥5)			
		Male		Female	
		aOR	95% CI	aOR	95% CI
	Never-smoker	1.00		1.00	
	Current smoker	1.36	(1.29−1.43)	1.97	(1.83−2.11)
Pack-year	Former smoker (pack-year ~ 10)	1.07	(0.99−1.15)	1.59	(1.46−1.74)
	Former smoker (pack-year 10~20)	1.12	(1.03−1.21)	2.06	(1.65−2.57)
	Former smoker (pack-year 20~30)	1.17	(1.07−1.28)	1.53	(1.02−2.29)
	Former smoker (pack-year 30~)	1.36	(1.27−1.46)	1.50	(1.02−2.21)
Former tobacco type	Former smoker (CC only)	1.18	(1.11−1.25)	1.61	(1.48−1.76)
	Former smoker (EC only)	1.44	(0.91−2.31)	1.91	(1.29−2.84)
	Former smoker (EC&CC)	1.51	(1.33−1.72)	1.91	(1.56−2.33)

aOR, adjusted odds ratio; CI, confidence interval.

Additionally, the aORs increased progressively with higher pack-years among men, indicating a positive dose–response relationship between cumulative smoking exposure and depressive symptoms. However, in women, the number of participants with ≥20 pack-years was relatively small, and no statistically significant trend was observed.

The association between smoking status and the severity of depressive symptoms was also analyzed (Table 5). Among men, former smokers showed higher aORs for mild and moderate depression (aOR: 1.18, 95% CI: 1.11–1.25 for mild depression; aOR: 1.19, 95% CI: 1.06–1.33, for moderate depression; aOR: 1.10, 95% CI: 0.79–1.53 for severe depression), whereas the aORs among women were higher for moderate and severe depression (aOR: 1.55, 95% CI: 1.42–1.69 for mild depression; aOR: 2.02, 95% CI: 1.78–2.31 for moderate depression; aOR: 2.08, 95% CI: 1.48–2.93 for severe depression).

**Table 5:** Adjusted odds ratios for depressive symptoms according to smoking status, stratified by depression severity.

Variables	Male						
	Depressive symptoms						
	None	Mild (5 ≤ PHQ-9 < 9)		Moderate (10 ≤ PHQ-9 < 20)		Severe (20 ≤ PHQ-9)	
	OR	OR	95% CI	OR	95% CI	OR	95% CI
Smoking status							
Never-smoker	1.00						
Current smoker		1.33	(1.26–1.41)	1.52	(1.36–1.70)	1.25	(0.91–1.73)
Former smoker		1.18	(1.11−1.25)	1.19	(1.06–1.33)	1.10	(0.79–1.53)
Female							
Never-smoker	1.00						
Current smoker		1.74	(1.61−1.88)	2.79	(2.50–3.11)	2.93	(2.22–3.88)
Former smoker		1.55	(1.42−1.69)	2.02	(1.78–2.31)	2.08	(1.48–2.93)

aOR, adjusted odds ratio; CI, confidence interval.

## 4 Discussion

### 4.1 Principal findings

This study revealed that former smokers had lower odds of depressive symptoms compared with current smokers, although their odds remained higher than those of never smokers. This pattern is consistent with prior meta-analyses and cohort evidence suggesting that depressive symptoms tend to improve after smoking cessation (4, 15). However, the benefits of cessation were not universal; subgroup analyses of former smokers showed elevated odds of depressive symptoms among women, individuals with high cumulative exposure, recent quitters, and former dual users. These subgroup-specific patterns warrant cautious interpretation, as our analyses were primarily descriptive and not designed to formally test differences across these characteristics.

### 4.2 Biological and clinical explanations

Biologically, more depressive symptoms in current smokers can be explained by chronic nicotine exposure. Chronic nicotine desensitizes and upregulates nicotinic acetylcholine receptors and disrupts dopaminergic and serotonergic signaling, ultimately worsening depressive symptoms (16). In contrast, smoking cessation may interrupt the withdrawal–relief cycle and allow neurobiological recalibration, which is associated with reduced depressive symptoms (17). Cessation also attenuates hypothalamic–pituitary–adrenal axis hyper reactivity and reduces systemic inflammation (e.g., CRP and IL-6), which are changes compatible with mood improvement (18, 19). However, our research suggests that these mental health benefits may not be universally observed in the vulnerable subgroups, indicating a residual risk despite abstinence.

### 4.3 Vulnerable subgroups

We identified a dose–response relationship in this study, wherein former smokers with a history of high cumulative exposure (i.e., high pack-years) demonstrated significantly greater odds of depressive symptoms than those with lower lifetime exposure. This finding can be explained by higher nicotine dependence, which results in greater upregulation of nicotinic acetylcholine receptors and more substantial dysregulation of the dopaminergic and serotonergic pathways that are critical for mood regulation. Additionally, a high pack-year history is unequivocally linked to a greater burden of smoking-related chronic diseases, such as cardiovascular disease and COPD, as well as subclinical systemic inflammation. The presence of chronic physical comorbidities is a powerful independent risk factor for depression. Therefore, the elevated odds of depressive symptoms observed in high-exposure former smokers may reflect not only the lasting neurobiological toll of heavy smoking but also the concurrent mental health consequences of smoking-induced physical morbidity.

In this study, women who were either current or former smokers appeared to have higher adjusted odds of depressive symptoms than men, which is consistent with previous literature (20). Several mechanisms may help explain this pattern. Women generally have a higher baseline prevalence of depressive disorders and may exhibit greater sensitivity to stress (21). In addition, fluctuations in reproductive hormones across the menstrual cycle, pregnancy, and the menopausal transition may influence mood through neuroendocrine pathways (22). Smoking motives also differ by sex; women more often report smoking for stress relief and mood regulation, which may strengthen the link between smoking and depressive symptoms (23). Collectively, these findings suggest that women may represent a subgroup that requires particular clinical attention during cessation, although further studies are needed to confirm whether these differences reflect true sex-specific effects.

Cessation duration was also an important factor in this study, as another vulnerable subgroup of former smokers was recent quitters. Recent quitters (<1 year) not only had the highest odds of depressive symptoms among former smokers but also exhibited even higher risks than current smokers. However, as the cessation duration increased, depressive symptoms dropped below current-smoker levels. This increase in depression among recent quitters is consistent with withdrawal-related mood instability early in cessation. A plausible biological explanation is that early abstinence removes nicotine as an activator of nicotinic acetylcholine receptors and monoamine oxidase inhibition, which can temporarily lower dopamine and serotonin levels and worsen depressive symptoms (24). Over the following months, neuroadaptation proceeds, hypothalamic–pituitary–adrenal axis hyperreactivity normalizes, and systemic inflammation declines, ultimately improving depressive symptoms (17). Our research is consistent with longitudinal studies reporting early mood worsening around the time of quitting but improvements by 6–12 months relative to continuing smokers (25). Clinically, this study highlights the need for proactive mood monitoring and targeted psychosocial support during the first year of tobacco abstinence.

Finally, the type of tobacco products used by former smokers appears to be relevant to depression. Former smokers with a history of dual use of conventional cigarettes and e-cigarettes had higher odds of developing depressive symptoms than those who used conventional cigarettes only. To our knowledge, few studies have specifically compared depressive symptoms among former smokers based on their prior product history. Nevertheless, a consistent body of evidence shows that current dual users have worse mental health profiles than current exclusive cigarette smokers and never smokers, including higher odds of experiencing depressive symptoms and psychological distress (26–28). Dual use is associated with greater nicotine exposure and dependence than cigarette-only use, which potentially compounds mood vulnerability (27). These patterns suggest that former smokers with a history of dual use may remain at a heightened risk, which is clinically relevant. Notably, many smokers have transitioned from conventional cigarettes to e-cigarettes in an attempt to quit smoking. While some research indicates that e-cigarettes can increase cessation rates compared to non-nicotine e-cigarettes and other nicotine replacement therapies (29), our research suggests that the mental health impact of switching might not be uniformly beneficial. Therefore, further research is needed to clarify the mental health effects of switching from conventional cigarettes to e-cigarettes. Additionally, clinicians may reasonably prioritize complete abstinence from conventional cigarettes and seek to minimize sustained dual use through routine screening for residual or emergent mood symptoms for cessation support.

### 4.4 Clinical implications

Taken together, our findings suggest that certain subgroups of former smokers may experience higher levels of depressive symptoms. However, given the cross-sectional design of the present study, these findings should be interpreted as hypothesis-generating rather than definitive evidence for clinical intervention strategies. If confirmed in longitudinal and interventional studies, these findings may support more tailored cessation support strategies, including closer mood monitoring and additional psychosocial support during the early abstinence period. Such an approach may help address the short-term vulnerability observed during early abstinence while supporting sustained cessation. Therefore, a clinical goal is to bridge the short-term vulnerability period so that long-term benefits can be realized (30).

### 4.5 Limitations

This study has certain intrinsic limitations. First, it was a cross-sectional study; therefore, the causal relationship between smoking cessation and depressive symptoms could not be clearly identified. Furthermore, instead of smoking cessation relieving depressive symptoms, the reverse is possible—individuals with fewer depressive symptoms may be more inclined to quit and sustain abstinence, causing recent quitters to appear more depressed (31). Therefore, longitudinal studies with repeated measurements of mood across cessation trajectories are required to establish causality.

Second, the KCHS classifies current and former smokers using self-reporting measures, which are susceptible to social desirability misclassification. This concern is especially striking for women: multiple studies show substantial underreporting of smoking among women when self-reports are compared with urinary cotinine or other biochemical markers (colloquially referred to as “hidden” female smokers) (32, 33). These misclassifications could bias associations between smoking status and depressive symptoms; in particular, respondents who minimize smoking are also more likely to underreport mood symptoms due to social desirability tendencies, potentially attenuating or distorting true effect estimates (34). While our 2024 data did not include biomarker data related to smoking, biochemical verification should be incorporated when feasible to improve the classification in future research. Urinary cotinine, the preferred biomarker of nicotine exposure, can detect undisclosed current smoking status over the previous 2–3 days (35). This approach could be particularly valuable for accurately identifying female smokers in settings with high stigma. However, cotinine will not reliably detect individuals who truly stopped smoking several days earlier (“hidden” former smokers who have been abstinent long enough for cotinine to clear). In such cases, longer-window biomarkers, such as urinary NNAL, can remain detectable for 6–12 weeks after exposure and may help distinguish recent former smokers (35).

Third, although we conducted an interaction test between sex and smoking status, this study was not designed to comprehensively evaluate effect modification across multiple participant characteristics. While sex-stratified analyses suggested differences in the magnitude of associations between men and women, these findings should be interpreted cautiously. Our analyses primarily aimed to describe subgroup patterns rather than formally test multiple interaction effects. Future studies with larger samples and adequate statistical power are needed to rigorously evaluate whether the associations between smoking cessation and depressive symptoms differ by sex, age, or other key characteristics.

Fourth, there were several limitations in the data variables. The variables of smoking status did not measure cessation duration at a finer resolution within the first year; therefore, we defined ‘recent quitters’ as <12 months since cessation. Although many cessation trials and frameworks define success as 6 or 12 months of abstinence, including the World Health Organization, which treats ≥6 months of abstinence as a stringent success endpoint, our data could not segment the 12-month window (36). In future research, the cessation duration should be reported in month-level periods and modeled as a time-varying exposure. These enhancements would allow alignment with the World Health Organization reporting standards (6 or 12-month abstinence endpoints), facilitating sensitivity analyses across multiple cessation windows to help recent quitters as a vulnerable group. Additionally, the study lacked information on several potentially important confounders, including psychiatric history, antidepressant medication use, and other substance use. The absence of these variables may result in residual confounding. Furthermore, cessation duration was self-reported and therefore subject to recall bias. Misclassification of smoking status may also be particularly relevant among women due to social desirability bias, which has been reported in prior studies of smoking behavior in East Asian populations.

Finally, the data on former e-cigarette use were incomplete, as past smoking duration, cumulative smoking exposure, and cessation duration were unavailable for e-cigarettes. Future studies should incorporate a dedicated e-cigarette module with standardized exposure metrics (e.g., device type, nicotine strength, and volume/frequency of use) and cessation duration data to enable dose–response modeling and better characterize depression risk among former dual users, a key vulnerable subgroup. These enhancements would yield more reliable dose–response estimates across conventional and non-conventional products and inform tailored cessation strategies to improve depressive symptoms in former dual users.

## 5 Conclusion

In conclusion, this study examined the association between smoking cessation and the prevalence of depressive symptoms. Although former smokers showed lower odds of depressive symptoms than current smokers, certain subgroups—including women, individuals with high cumulative exposure, recent quitters, and dual users—showed comparatively higher odds.

These findings suggest that vulnerability to depressive symptoms may differ across subgroups of former smokers. Future longitudinal studies are needed to clarify causality and to identify appropriate cessation and mental health support strategies for groups at potentially higher risk.

## Data Availability

Publicly available datasets were analyzed in this study. This data can be found at: https://chs.kdca.go.kr/chs/main.do.
