# Supplementary material for: Association between smoking cessation and depressive symptoms according to cessation duration, pack-years, and tobacco product type: a nationwide cross-sectional study in Korea
Source: Front Public Health. 2026 Mar 26;14:1755259. doi: 10.3389/fpubh.2026.1755259 (PMC13100510; doi:10.3389/fpubh.2026.1755259)
Supplement: Supplementary file 1 [file Table_1.pdf]

Supplementary table S1. Joint test for interaction between sex and lifetime smoking status in survey-weighted logistic regression

Effect	Num DF	Den DF	F value	p value
**Sex × smoking status**	2	227293	55.03	<.0001

P values were derived from joint tests of the sex × smoking status interaction
