# Supplementary material for: Association between smoking cessation and depressive symptoms according to cessation duration, pack-years, and tobacco product type: a nationwide cross-sectional study in Korea
Source: Front Public Health. 2026 Mar 26;14:1755259. doi: 10.3389/fpubh.2026.1755259 (PMC13100510; doi:10.3389/fpubh.2026.1755259)

Supplementary table S2. Directed Acyclic Graph (DAG) depicting the hypothesized causal structure of the association between smoking status and depressive symptoms, with perceived stress treated as a confounder

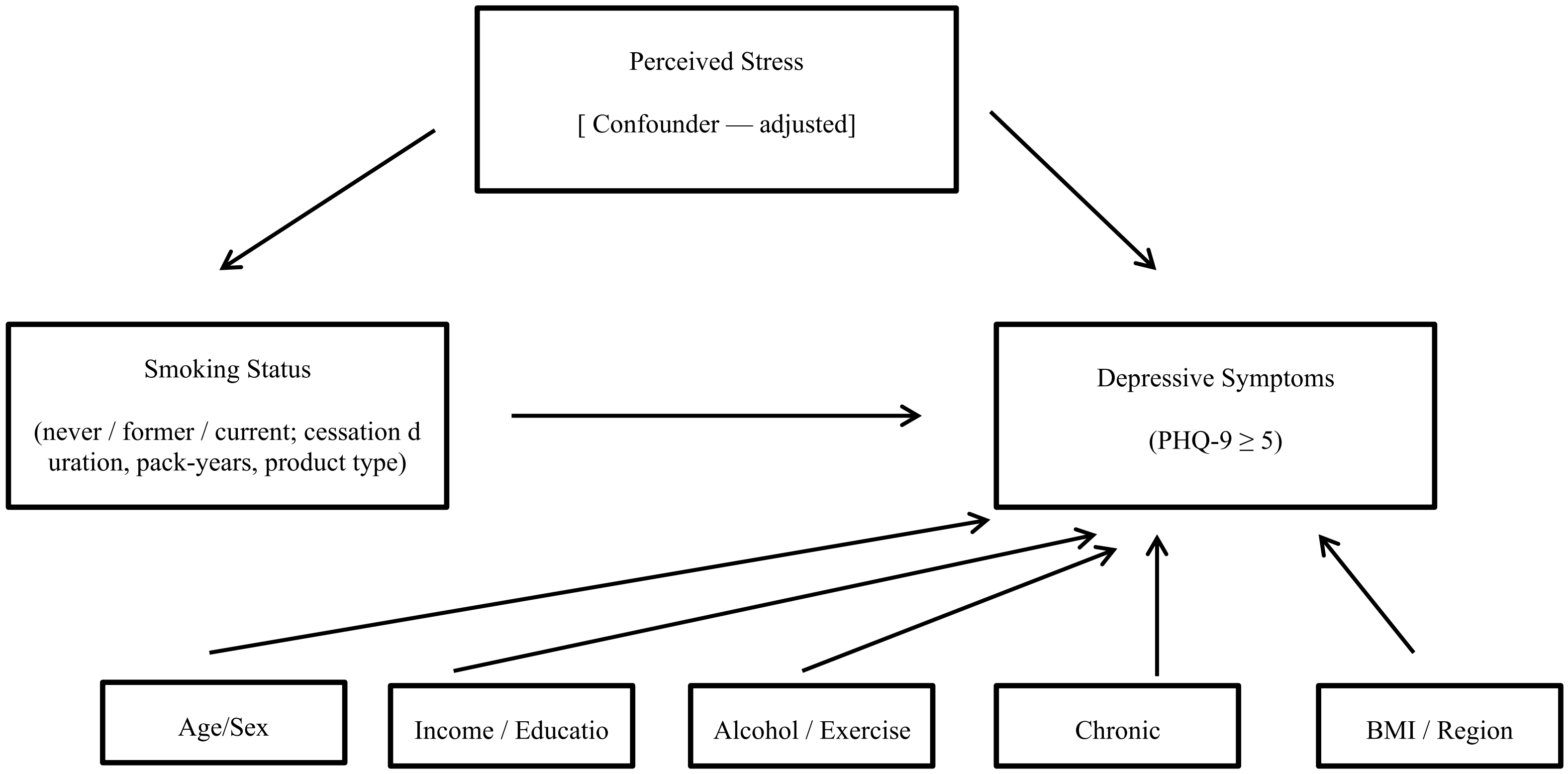

Supplement: Supplementary file 2 [file Table_2.pdf]
